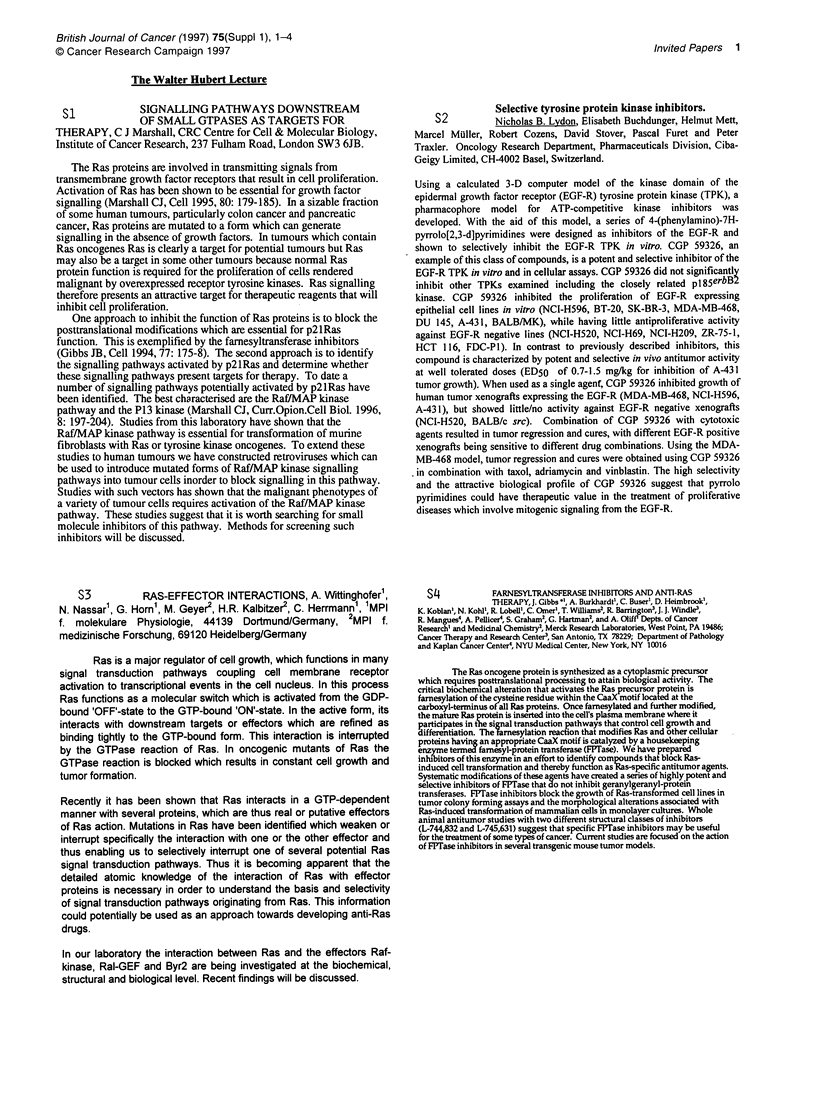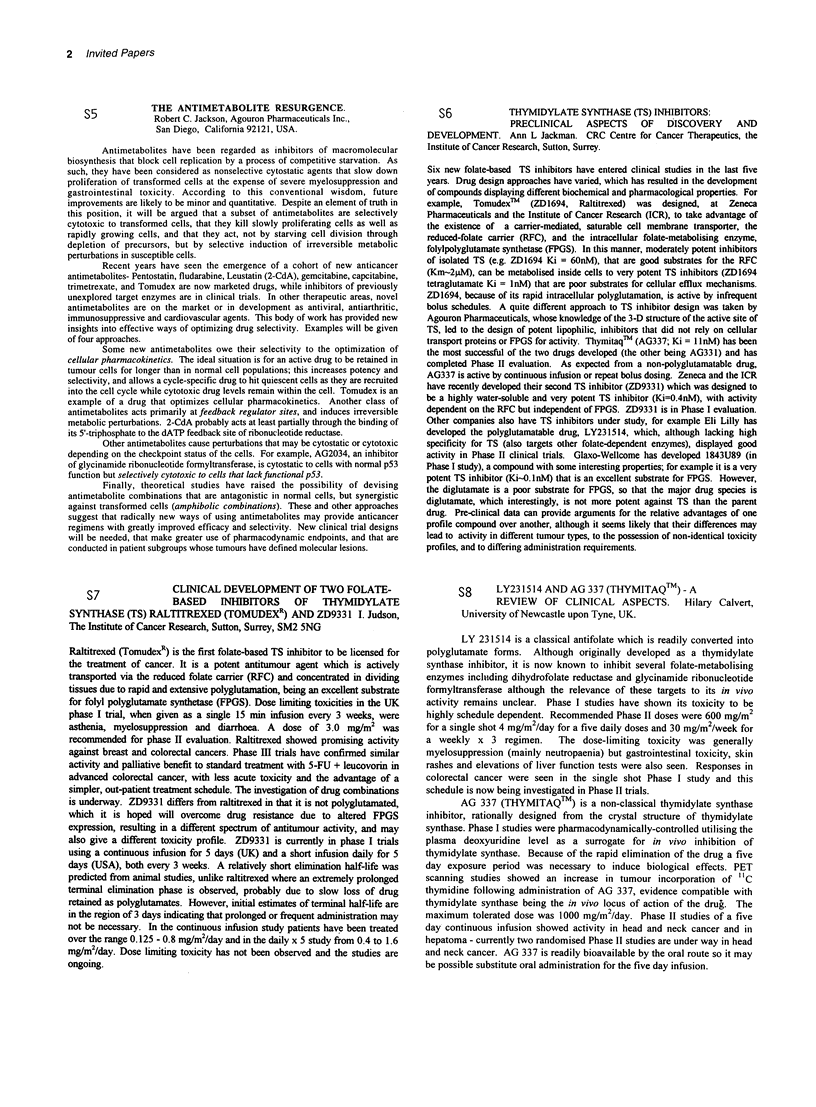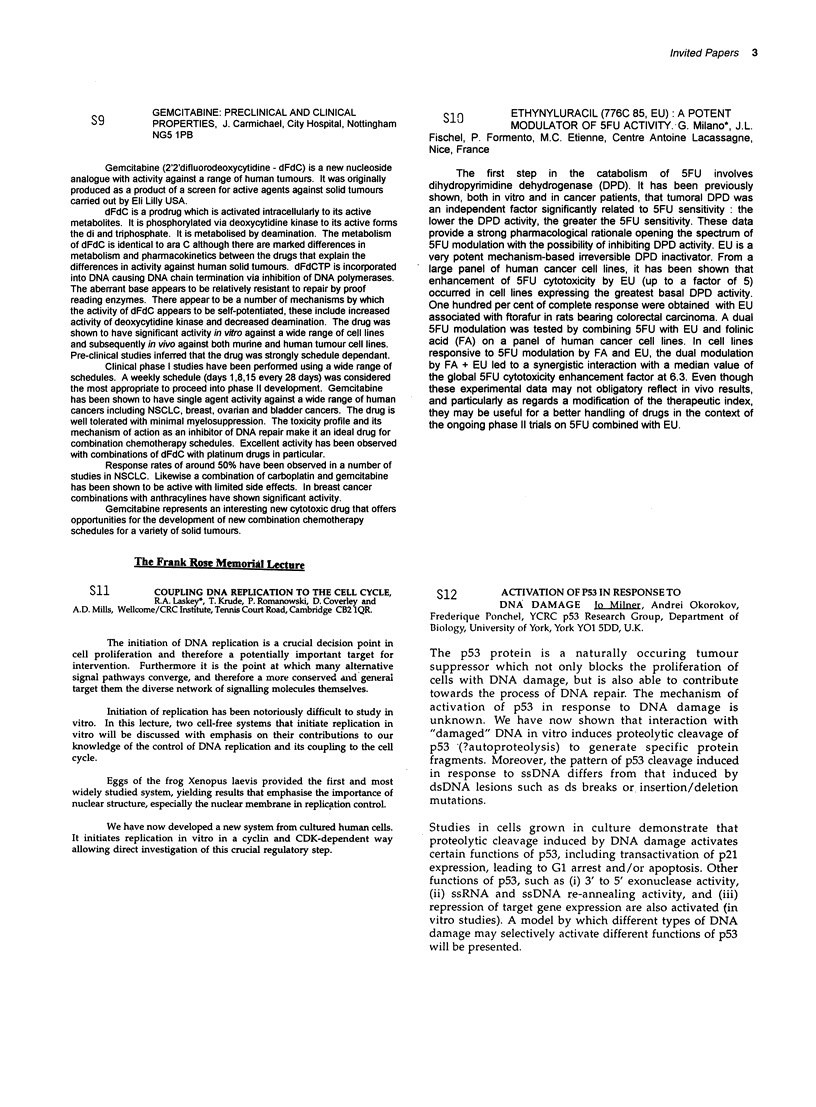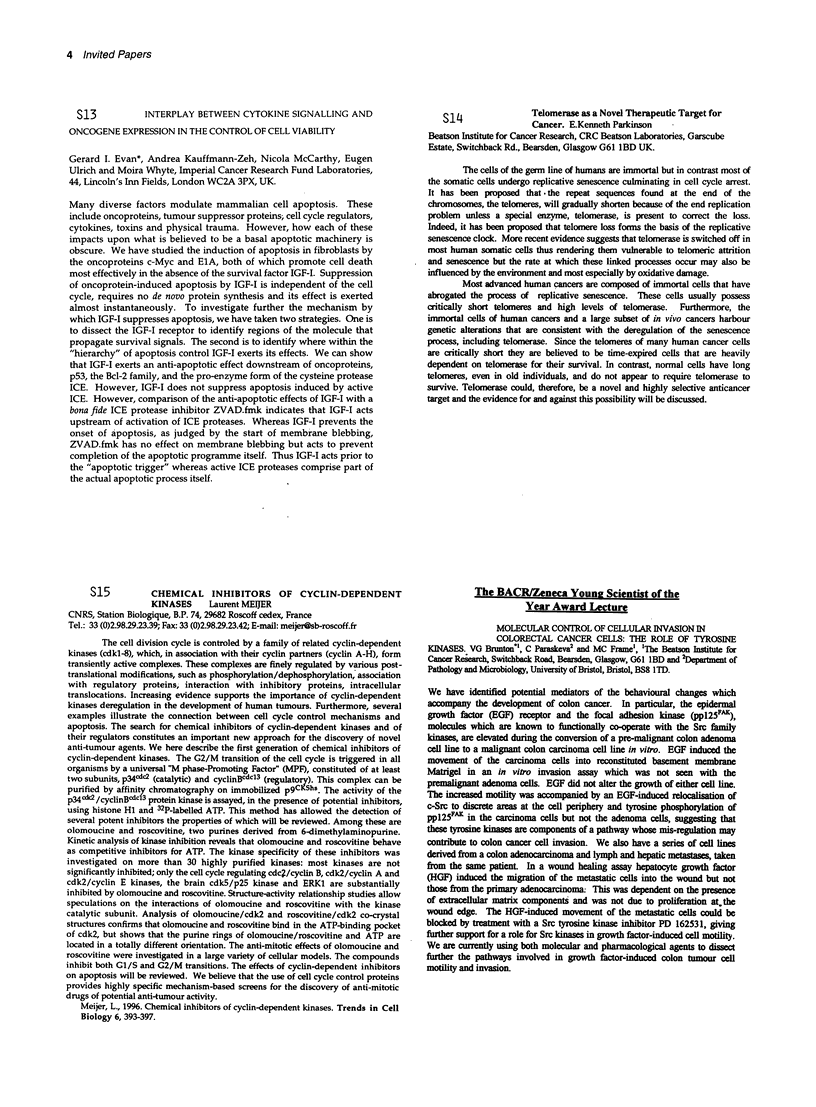# British Association for Cancer Research 38th annual meeting. New Targets in Cancer Chemotherapy Southampton, United Kingdom, 1-4 April 1997. Abstracts.

**Published:** 1997

**Authors:** 


British Journal of Cancer (1997) 75(Suppl 1), 1–4
© Cancer Research Campaign 1997

**The Walter Hubert Lecture**

S1 SIGNALLING PATHWAYS DOWNSTREAM OF SMALL GTPASES AS TARGETS FOR THERAPY, C J Marshall, CRC Centre for Cell & Molecular Biology, Institute of Cancer Research, 237 Fulham Road, London SW3 6JB.

The Ras proteins are involved in transmitting signals from transmembrane growth factor receptors that result in cell proliferation. Activation of Ras has been shown to be essential for growth factor signalling (Marshall CJ, Cell 1995, 80: 179-185). In a sizable fraction of some human tumours, particularly colon cancer and pancreatic cancer, Ras proteins are mutated to a form which can generate signalling in the absence of growth factors. In tumours which contain Ras oncogenes Ras is clearly a target for potential tumours but Ras may also be a target in some other tumours because normal Ras protein function is required for the proliferation of cells rendered malignant by overexpressed receptor tyrosine kinases. Ras signalling therefore presents an attractive target for therapeutic reagents that will inhibit cell proliferation.

One approach to inhibit the function of Ras proteins is to block the posttranslational modifications which are essential for p21Ras function. This is exemplified by the farnesyltransferase inhibitors (Gibbs JB, Cell 1994, 77: 175-8). The second approach is to identify the signalling pathways activated by p21Ras and determine whether these signalling pathways present targets for therapy. To date a number of signalling pathways potentially activated by p21Ras have been identified. The best characterised are the Raf/MAP kinase pathway and the P13 kinase (Marshall CJ, Curr.Opion.Cell Biol. 1996, 8: 197-204). Studies from this laboratory have shown that the Raf/MAP kinase pathway is essential for transformation of murine fibroblasts with Ras or tyrosine kinase oncogenes. To extend these studies to human tumours we have constructed retroviruses which can be used to introduce mutated forms of Raf/MAP kinase signalling pathways into tumour cells inorder to block signalling in this pathway. Studies with such vectors has shown that the malignant phenotypes of a variety of tumour cells requires activation of the Raf/MAP kinase pathway. These studies suggest that it is worth searching for small molecule inhibitors of this pathway. Methods for screening such inhibitors will be discussed.

S2 **Selective tyrosine protein kinase inhibitors.**

Nicholas B. Lydon, Elisabeth Buchdunger, Helmut Mett, Marcel Müller, Robert Cozens, David Stover, Pascal Furet and Peter Traxler. Oncology Research Department, Pharmaceuticals Division, Ciba-Geigy Limited, CH-4002 Basel, Switzerland.

Using a calculated 3-D computer model of the kinase domain of the epidermal growth factor receptor (EGF-R) tyrosine protein kinase (TPK), a pharmacophore model for ATP-competitive kinase inhibitors was developed. With the aid of this model, a series of 4-(phenylamino)-7H-pyrrolo[2,3-d]pyrimidines were designed as inhibitors of the EGF-R and shown to selectively inhibit the EGF-R TPK *in vitro*. CGP 59326, an example of this class of compounds, is a potent and selective inhibitor of the EGF-R TPK *in vitro* and in cellular assays. CGP 59326 did not significantly inhibit other TPKs examined including the closely related p185$^{erbB2}$ kinase. CGP 59326 inhibited the proliferation of EGF-R expressing epithelial cell lines *in vitro* (NCI-H596, BT-20, SK-BR-3, MDA-MB-468, DU 145, A-431, BALB/MK), while having little antiproliferative activity against EGF-R negative lines (NCI-H520, NCI-H69, NCI-H209, ZR-75-1, HCT 116, FDC-P1). In contrast to previously described inhibitors, this compound is characterized by potent and selective *in vivo* antitumor activity at well tolerated doses ($ED_{50}$ of 0.7-1.5 mg/kg for inhibition of A-431 tumor growth). When used as a single agent, CGP 59326 inhibited growth of human tumor xenografts expressing the EGF-R (MDA-MB-468, NCI-H596, A-431), but showed little/no activity against EGF-R negative xenografts (NCI-H520, BALB/c *src*). Combination of CGP 59326 with cytotoxic agents resulted in tumor regression and cures, with different EGF-R positive xenografts being sensitive to different drug combinations. Using the MDA-MB-468 model, tumor regression and cures were obtained using CGP 59326 in combination with taxol, adriamycin and vinblastin. The high selectivity and the attractive biological profile of CGP 59326 suggest that pyrrolo pyrimidines could have therapeutic value in the treatment of proliferative diseases which involve mitogenic signaling from the EGF-R.

S3 RAS-EFFECTOR INTERACTIONS, A. Wittinghofer[1], N. Nassar[1], G. Horn[1], M. Geyer[2], H.R. Kalbitzer[2], C. Herrmann[1], [1]MPI f. molekulare Physiologie, 44139 Dortmund/Germany, [2]MPI f. medizinische Forschung, 69120 Heidelberg/Germany

Ras is a major regulator of cell growth, which functions in many signal transduction pathways coupling cell membrane receptor activation to transcriptional events in the cell nucleus. In this process Ras functions as a molecular switch which is activated from the GDP-bound 'OFF'-state to the GTP-bound 'ON'-state. In the active form, its interacts with downstream targets or effectors which are refined as binding tightly to the GTP-bound form. This interaction is interrupted by the GTPase reaction of Ras. In oncogenic mutants of Ras the GTPase reaction is blocked which results in constant cell growth and tumor formation.

Recently it has been shown that Ras interacts in a GTP-dependent manner with several proteins, which are thus real or putative effectors of Ras action. Mutations in Ras have been identified which weaken or interrupt specifically the interaction with one or the other effector and thus enabling us to selectively interrupt one of several potential Ras signal transduction pathways. Thus it is becoming apparent that the detailed atomic knowledge of the interaction of Ras with effector proteins is necessary in order to understand the basis and selectivity of signal transduction pathways originating from Ras. This information could potentially be used as an approach towards developing anti-Ras drugs.

In our laboratory the interaction between Ras and the effectors Raf-kinase, Ral-GEF and Byr2 are being investigated at the biochemical, structural and biological level. Recent findings will be discussed.

S4 FARNESYLTRANSFERASE INHIBITORS AND ANTI-RAS THERAPY, J. Gibbs *[1], A. Burkhardt[1], C. Buser[1], D. Heimbrook[1], K. Koblan[1], N. Kohl[1], R. Lobell[1], C. Omer[1], T. Williams[2], R. Barrington[3], J. J. Windle[3], R. Mangues[4], A. Pellicer[4], S. Graham[2], G. Hartman[2], and A. Oliff[1] Depts. of Cancer Research[1] and Medicinal Chemistry[2], Merck Research Laboratories, West Point, PA 19486; Cancer Therapy and Research Center[3], San Antonio, TX 78229; Department of Pathology and Kaplan Cancer Center[4], NYU Medical Center, New York, NY 10016

The Ras oncogene protein is synthesized as a cytoplasmic precursor which requires posttranslational processing to attain biological activity. The critical biochemical alteration that activates the Ras precursor protein is farnesylation of the cysteine residue within the CaaX motif located at the carboxyl-terminus of all Ras proteins. Once farnesylated and further modified, the mature Ras protein is inserted into the cell's plasma membrane where it participates in the signal transduction pathways that control cell growth and differentiation. The farnesylation reaction that modifies Ras and other cellular proteins having an appropriate CaaX motif is catalyzed by a housekeeping enzyme termed farnesyl-protein transferase (FPTase). We have prepared inhibitors of this enzyme in an effort to identify compounds that block Ras-induced cell transformation and thereby function as Ras-specific antitumor agents. Systematic modifications of these agents have created a series of highly potent and selective inhibitors of FPTase that do not inhibit geranylgeranyl-protein transferases. FPTase inhibitors block the growth of Ras-transformed cell lines in tumor colony forming assays and the morphological alterations associated with Ras-induced transformation of mammalian cells in monolayer cultures. Whole animal antitumor studies with two different structural classes of inhibitors (L-744,832 and L-745,631) suggest that specific FPTase inhibitors may be useful for the treatment of some types of cancer. Current studies are focused on the action of FPTase inhibitors in several transgenic mouse tumor models.

### S5 THE ANTIMETABOLITE RESURGENCE.

Robert C. Jackson, Agouron Pharmaceuticals Inc., San Diego, California 92121, USA.

Antimetabolites have been regarded as inhibitors of macromolecular biosynthesis that block cell replication by a process of competitive starvation. As such, they have been considered as nonselective cytostatic agents that slow down proliferation of transformed cells at the expense of severe myelosuppression and gastrointestinal toxicity. According to this conventional wisdom, future improvements are likely to be minor and quantitative. Despite an element of truth in this position, it will be argued that a subset of antimetabolites are selectively cytotoxic to transformed cells, that they kill slowly proliferating cells as well as rapidly growing cells, and that they act, not by starving cell division through depletion of precursors, but by selective induction of irreversible metabolic perturbations in susceptible cells.

Recent years have seen the emergence of a cohort of new anticancer antimetabolites- Pentostatin, fludarabine, Leustatin (2-CdA), gemcitabine, capcitabine, trimetrexate, and Tomudex are now marketed drugs, while inhibitors of previously unexplored target enzymes are in clinical trials. In other therapeutic areas, novel antimetabolites are on the market or in development as antiviral, antiarthritic, immunosuppressive and cardiovascular agents. This body of work has provided new insights into effective ways of optimizing drug selectivity. Examples will be given of four approaches.

Some new antimetabolites owe their selectivity to the optimization of *cellular pharmacokinetics*. The ideal situation is for an active drug to be retained in tumour cells for longer than in normal cell populations; this increases potency and selectivity, and allows a cycle-specific drug to hit quiescent cells as they are recruited into the cell cycle while cytotoxic drug levels remain within the cell. Tomudex is an example of a drug that optimizes cellular pharmacokinetics. Another class of antimetabolites acts primarily at *feedback regulator sites*, and induces irreversible metabolic perturbations. 2-CdA probably acts at least partially through the binding of its 5'-triphosphate to the dATP feedback site of ribonucleotide reductase.

Other antimetabolites cause perturbations that may be cytostatic or cytotoxic depending on the checkpoint status of the cells. For example, AG2034, an inhibitor of glycinamide ribonucleotide formyltransferase, is cytostatic to cells with normal p53 function but *selectively cytotoxic to cells that lack functional p53*.

Finally, theoretical studies have raised the possibility of devising antimetabolite combinations that are antagonistic in normal cells, but synergistic against transformed cells (*amphibolic combinations*). These and other approaches suggest that radically new ways of using antimetabolites may provide anticancer regimens with greatly improved efficacy and selectivity. New clinical trial designs will be needed, that make greater use of pharmacodynamic endpoints, and that are conducted in patient subgroups whose tumours have defined molecular lesions.

### S6 THYMIDYLATE SYNTHASE (TS) INHIBITORS: PRECLINICAL ASPECTS OF DISCOVERY AND DEVELOPMENT.

Ann L Jackman. CRC Centre for Cancer Therapeutics, the Institute of Cancer Research, Sutton, Surrey.

Six new folate-based TS inhibitors have entered clinical studies in the last five years. Drug design approaches have varied, which has resulted in the development of compounds displaying different biochemical and pharmacological properties. For example, Tomudex™ (ZD1694, Raltitrexed) was designed, at Zeneca Pharmaceuticals and the Institute of Cancer Research (ICR), to take advantage of the existence of a carrier-mediated, saturable cell membrane transporter, the reduced-folate carrier (RFC), and the intracellular folate-metabolising enzyme, folylpolyglutamate synthetase (FPGS). In this manner, moderately potent inhibitors of isolated TS (e.g. ZD1694 Ki = 60nM), that are good substrates for the RFC (Km~2μM), can be metabolised inside cells to very potent TS inhibitors (ZD1694 tetraglutamate Ki = 1nM) that are poor substrates for cellular efflux mechanisms. ZD1694, because of its rapid intracellular polyglutamation, is active by infrequent bolus schedules. A quite different approach to TS inhibitor design was taken by Agouron Pharmaceuticals, whose knowledge of the 3-D structure of the active site of TS, led to the design of potent lipophilic, inhibitors that did not rely on cellular transport proteins or FPGS for activity. Thymitaq™ (AG337; Ki = 11nM) has been the most successful of the two drugs developed (the other being AG331) and has completed Phase II evaluation. As expected from a non-polyglutamatable drug, AG337 is active by continuous infusion or repeat bolus dosing. Zeneca and the ICR have recently developed their second TS inhibitor (ZD9331) which was designed to be a highly water-soluble and very potent TS inhibitor (Ki=0.4nM), with activity dependent on the RFC but independent of FPGS. ZD9331 is in Phase I evaluation. Other companies also have TS inhibitors under study, for example Eli Lilly has developed the polyglutamatable drug, LY231514, which, although lacking high specificity for TS (also targets other folate-dependent enzymes), displayed good activity in Phase II clinical trials. Glaxo-Wellcome has developed 1843U89 (in Phase I study), a compound with some interesting properties; for example it is a very potent TS inhibitor (Ki~0.1nM) that is an excellent substrate for FPGS. However, the diglutamate is a poor substrate for FPGS, so that the major drug species is diglutamate, which interestingly, is not more potent against TS than the parent drug. Pre-clinical data can provide arguments for the relative advantages of one profile compound over another, although it seems likely that their differences may lead to activity in different tumour types, to the possession of non-identical toxicity profiles, and to differing administration requirements.

### S7 CLINICAL DEVELOPMENT OF TWO FOLATE-BASED INHIBITORS OF THYMIDYLATE SYNTHASE (TS) RALTITREXED (TOMUDEX[R]) AND ZD9331

I. Judson, The Institute of Cancer Research, Sutton, Surrey, SM2 5NG

Raltitrexed (Tomudex[R]) is the first folate-based TS inhibitor to be licensed for the treatment of cancer. It is a potent antitumour agent which is actively transported via the reduced folate carrier (RFC) and concentrated in dividing tissues due to rapid and extensive polyglutamation, being an excellent substrate for folyl polyglutamate synthetase (FPGS). Dose limiting toxicities in the UK phase I trial, when given as a single 15 min infusion every 3 weeks, were asthenia, myelosuppression and diarrhoea. A dose of 3.0 mg/m$^2$ was recommended for phase II evaluation. Raltitrexed showed promising activity against breast and colorectal cancers. Phase III trials have confirmed similar activity and palliative benefit to standard treatment with 5-FU + leucovorin in advanced colorectal cancer, with less acute toxicity and the advantage of a simpler, out-patient treatment schedule. The investigation of drug combinations is underway. ZD9331 differs from raltitrexed in that it is not polyglutamated, which it is hoped will overcome drug resistance due to altered FPGS expression, resulting in a different spectrum of antitumour activity, and may also give a different toxicity profile. ZD9331 is currently in phase I trials using a continuous infusion for 5 days (UK) and a short infusion daily for 5 days (USA), both every 3 weeks. A relatively short elimination half-life was predicted from animal studies, unlike raltitrexed where an extremely prolonged terminal elimination phase is observed, probably due to slow loss of drug retained as polyglutamates. However, initial estimates of terminal half-life are in the region of 3 days indicating that prolonged or frequent administration may not be necessary. In the continuous infusion study patients have been treated over the range 0.125 - 0.8 mg/m$^2$/day and in the daily x 5 study from 0.4 to 1.6 mg/m$^2$/day. Dose limiting toxicity has not been observed and the studies are ongoing.

### S8 LY231514 AND AG 337 (THYMITAQ™) - A REVIEW OF CLINICAL ASPECTS.

Hilary Calvert, University of Newcastle upon Tyne, UK.

LY 231514 is a classical antifolate which is readily converted into polyglutamate forms. Although originally developed as a thymidylate synthase inhibitor, it is now known to inhibit several folate-metabolising enzymes including dihydrofolate reductase and glycinamide ribonucleotide formyltransferase although the relevance of these targets to its *in vivo* activity remains unclear. Phase I studies have shown its toxicity to be highly schedule dependent. Recommended Phase II doses were 600 mg/m$^2$ for a single shot 4 mg/m$^2$/day for a five daily doses and 30 mg/m$^2$/week for a weekly x 3 regimen. The dose-limiting toxicity was generally myelosuppression (mainly neutropaenia) but gastrointestinal toxicity, skin rashes and elevations of liver function tests were also seen. Responses in colorectal cancer were seen in the single shot Phase I study and this schedule is now being investigated in Phase II trials.

AG 337 (THYMITAQ™) is a non-classical thymidylate synthase inhibitor, rationally designed from the crystal structure of thymidylate synthase. Phase I studies were pharmacodynamically-controlled utilising the plasma deoxyuridine level as a surrogate for *in vivo* inhibition of thymidylate synthase. Because of the rapid elimination of the drug a five day exposure period was necessary to induce biological effects. PET scanning studies showed an increase in tumour incorporation of $^{11}C$ thymidine following administration of AG 337, evidence compatible with thymidylate synthase being the *in vivo* locus of action of the drug. The maximum tolerated dose was 1000 mg/m$^2$/day. Phase II studies of a five day continuous infusion showed activity in head and neck cancer and in hepatoma - currently two randomised Phase II studies are under way in head and neck cancer. AG 337 is readily bioavailable by the oral route so it may be possible substitute oral administration for the five day infusion.

S9 GEMCITABINE: PRECLINICAL AND CLINICAL PROPERTIES, J. Carmichael, City Hospital, Nottingham NG5 1PB

Gemcitabine (2'2'difluorodeoxycytidine - dFdC) is a new nucleoside analogue with activity against a range of human tumours. It was originally produced as a product of a screen for active agents against solid tumours carried out by Eli Lilly USA.

dFdC is a prodrug which is activated intracellularly to its active metabolites. It is phosphorylated via deoxycytidine kinase to its active forms the di and triphosphate. It is metabolised by deamination. The metabolism of dFdC is identical to ara C although there are marked differences in metabolism and pharmacokinetics between the drugs that explain the differences in activity against human solid tumours. dFdCTP is incorporated into DNA causing DNA chain termination via inhibition of DNA polymerases. The aberrant base appears to be relatively resistant to repair by proof reading enzymes. There appear to be a number of mechanisms by which the activity of dFdC appears to be self-potentiated, these include increased activity of deoxycytidine kinase and decreased deamination. The drug was shown to have significant activity *in vitro* against a wide range of cell lines and subsequently *in vivo* against both murine and human tumour cell lines. Pre-clinical studies inferred that the drug was strongly schedule dependant.

Clinical phase I studies have been performed using a wide range of schedules. A weekly schedule (days 1,8,15 every 28 days) was considered the most appropriate to proceed into phase II development. Gemcitabine has been shown to have single agent activity against a wide range of human cancers including NSCLC, breast, ovarian and bladder cancers. The drug is well tolerated with minimal myelosuppression. The toxicity profile and its mechanism of action as an inhibitor of DNA repair make it an ideal drug for combination chemotherapy schedules. Excellent activity has been observed with combinations of dFdC with platinum drugs in particular.

Response rates of around 50% have been observed in a number of studies in NSCLC. Likewise a combination of carboplatin and gemcitabine has been shown to be active with limited side effects. In breast cancer combinations with anthracylines have shown significant activity.

Gemcitabine represents an interesting new cytotoxic drug that offers opportunities for the development of new combination chemotherapy schedules for a variety of solid tumours.

S10 ETHYNYLURACIL (776C 85, EU) : A POTENT MODULATOR OF 5FU ACTIVITY. G. Milano*, J.L. Fischel, P. Formento, M.C. Etienne, Centre Antoine Lacassagne, Nice, France

The first step in the catabolism of 5FU involves dihydropyrimidine dehydrogenase (DPD). It has been previously shown, both in vitro and in cancer patients, that tumoral DPD was an independent factor significantly related to 5FU sensitivity : the lower the DPD activity, the greater the 5FU sensitivity. These data provide a strong pharmacological rationale opening the spectrum of 5FU modulation with the possibility of inhibiting DPD activity. EU is a very potent mechanism-based irreversible DPD inactivator. From a large panel of human cancer cell lines, it has been shown that enhancement of 5FU cytotoxicity by EU (up to a factor of 5) occurred in cell lines expressing the greatest basal DPD activity. One hundred per cent of complete response were obtained with EU associated with ftorafur in rats bearing colorectal carcinoma. A dual 5FU modulation was tested by combining 5FU with EU and folinic acid (FA) on a panel of human cancer cell lines. In cell lines responsive to 5FU modulation by FA and EU, the dual modulation by FA + EU led to a synergistic interaction with a median value of the global 5FU cytotoxicity enhancement factor at 6.3. Even though these experimental data may not obligatory reflect in vivo results, and particularly as regards a modification of the therapeutic index, they may be useful for a better handling of drugs in the context of the ongoing phase II trials on 5FU combined with EU.

### The Frank Rose Memorial Lecture

S11 **COUPLING DNA REPLICATION TO THE CELL CYCLE,** R.A. Laskey*, T. Krude, P. Romanowski, D. Coverley and A.D. Mills, Wellcome/CRC Institute, Tennis Court Road, Cambridge CB2 1QR.

The initiation of DNA replication is a crucial decision point in cell proliferation and therefore a potentially important target for intervention. Furthermore it is the point at which many alternative signal pathways converge, and therefore a more conserved and general target them the diverse network of signalling molecules themselves.

Initiation of replication has been notoriously difficult to study in vitro. In this lecture, two cell-free systems that initiate replication in vitro will be discussed with emphasis on their contributions to our knowledge of the control of DNA replication and its coupling to the cell cycle.

Eggs of the frog Xenopus laevis provided the first and most widely studied system, yielding results that emphasise the importance of nuclear structure, especially the nuclear membrane in replication control.

We have now developed a new system from cultured human cells. It initiates replication in vitro in a cyclin and CDK-dependent way allowing direct investigation of this crucial regulatory step.

S12 ACTIVATION OF P53 IN RESPONSE TO DNA DAMAGE Jo Milner, Andrei Okorokov, Frederique Ponchel, YCRC p53 Research Group, Department of Biology, University of York, York YO1 5DD, U.K.

The p53 protein is a naturally occuring tumour suppressor which not only blocks the proliferation of cells with DNA damage, but is also able to contribute towards the process of DNA repair. The mechanism of activation of p53 in response to DNA damage is unknown. We have now shown that interaction with "damaged" DNA in vitro induces proteolytic cleavage of p53 (?autoproteolysis) to generate specific protein fragments. Moreover, the pattern of p53 cleavage induced in response to ssDNA differs from that induced by dsDNA lesions such as ds breaks or insertion/deletion mutations.

Studies in cells grown in culture demonstrate that proteolytic cleavage induced by DNA damage activates certain functions of p53, including transactivation of p21 expression, leading to G1 arrest and/or apoptosis. Other functions of p53, such as (i) 3' to 5' exonuclease activity, (ii) ssRNA and ssDNA re-annealing activity, and (iii) repression of target gene expression are also activated (in vitro studies). A model by which different types of DNA damage may selectively activate different functions of p53 will be presented.

S13 INTERPLAY BETWEEN CYTOKINE SIGNALLING AND ONCOGENE EXPRESSION IN THE CONTROL OF CELL VIABILITY

Gerard I. Evan*, Andrea Kauffmann-Zeh, Nicola McCarthy, Eugen Ulrich and Moira Whyte, Imperial Cancer Research Fund Laboratories, 44, Lincoln's Inn Fields, London WC2A 3PX, UK.

Many diverse factors modulate mammalian cell apoptosis. These include oncoproteins, tumour suppressor proteins, cell cycle regulators, cytokines, toxins and physical trauma. However, how each of these impacts upon what is believed to be a basal apoptotic machinery is obscure. We have studied the induction of apoptosis in fibroblasts by the oncoproteins c-Myc and E1A, both of which promote cell death most effectively in the absence of the survival factor IGF-I. Suppression of oncoprotein-induced apoptosis by IGF-I is independent of the cell cycle, requires no *de novo* protein synthesis and its effect is exerted almost instantaneously. To investigate further the mechanism by which IGF-I suppresses apoptosis, we have taken two strategies. One is to dissect the IGF-I receptor to identify regions of the molecule that propagate survival signals. The second is to identify where within the "hierarchy" of apoptosis control IGF-I exerts its effects. We can show that IGF-I exerts an anti-apoptotic effect downstream of oncoproteins, p53, the Bcl-2 family, and the pro-enzyme form of the cysteine protease ICE. However, IGF-I does not suppress apoptosis induced by active ICE. However, comparison of the anti-apoptotic effects of IGF-I with a *bona fide* ICE protease inhibitor ZVAD.fmk indicates that IGF-I acts upstream of activation of ICE proteases. Whereas IGF-I prevents the onset of apoptosis, as judged by the start of membrane blebbing, ZVAD.fmk has no effect on membrane blebbing but acts to prevent completion of the apoptotic programme itself. Thus IGF-I acts prior to the "apoptotic trigger" whereas active ICE proteases comprise part of the actual apoptotic process itself.

S14 **Telomerase as a Novel Therapeutic Target for Cancer.** E.Kenneth Parkinson

Beatson Institute for Cancer Research, CRC Beatson Laboratories, Garscube Estate, Switchback Rd., Bearsden, Glasgow G61 1BD UK.

The cells of the germ line of humans are immortal but in contrast most of the somatic cells undergo replicative senescence culminating in cell cycle arrest. It has been proposed that the repeat sequences found at the end of the chromosomes, the telomeres, will gradually shorten because of the end replication problem unless a special enzyme, telomerase, is present to correct the loss. Indeed, it has been proposed that telomere loss forms the basis of the replicative senescence clock. More recent evidence suggests that telomerase is switched off in most human somatic cells thus rendering them vulnerable to telomeric attrition and senescence but the rate at which these linked processes occur may also be influenced by the environment and most especially by oxidative damage.

Most advanced human cancers are composed of immortal cells that have abrogated the process of replicative senescence. These cells usually possess critically short telomeres and high levels of telomerase. Furthermore, the immortal cells of human cancers and a large subset of *in vivo* cancers harbour genetic alterations that are consistent with the deregulation of the senescence process, including telomerase. Since the telomeres of many human cancer cells are critically short they are believed to be time-expired cells that are heavily dependent on telomerase for their survival. In contrast, normal cells have long telomeres, even in old individuals, and do not appear to require telomerase to survive. Telomerase could, therefore, be a novel and highly selective anticancer target and the evidence for and against this possibility will be discussed.

S15 CHEMICAL INHIBITORS OF CYCLIN-DEPENDENT KINASES Laurent MEIJER

CNRS, Station Biologique, B.P. 74, 29682 Roscoff cedex, France
Tel.: 33 (0)2.98.29.23.39; Fax: 33 (0)2.98.29.23.42; E-mail: meijer@sb-roscoff.fr

The cell division cycle is controled by a family of related cyclin-dependent kinases (cdk1-8), which, in association with their cyclin partners (cyclin A-H), form transiently active complexes. These complexes are finely regulated by various post-translational modifications, such as phosphorylation/dephosphorylation, association with regulatory proteins, interaction with inhibitory proteins, intracellular translocations. Increasing evidence supports the importance of cyclin-dependent kinases deregulation in the development of human tumours. Furthermore, several examples illustrate the connection between cell cycle control mechanisms and apoptosis. The search for chemical inhibitors of cyclin-dependent kinases and of their regulators constitutes an important new approach for the discovery of novel anti-tumour agents. We here describe the first generation of chemical inhibitors of cyclin-dependent kinases. The G2/M transition of the cell cycle is triggered in all organisms by a universal "M phase-Promoting Factor" (MPF), constituted of at least two subunits, $p34^{cdc2}$ (catalytic) and $cyclinB^{cdc13}$ (regulatory). This complex can be purified by affinity chromatography on immobilized $p9^{CKShs}$. The activity of the $p34^{cdc2}$/$cyclinB^{cdc13}$ protein kinase is assayed, in the presence of potential inhibitors, using histone H1 and $^{32}P$-labelled ATP. This method has allowed the detection of several potent inhibitors the properties of which will be reviewed. Among these are olomoucine and roscovitine, two purines derived from 6-dimethylaminopurine. Kinetic analysis of kinase inhibition reveals that olomoucine and roscovitine behave as competitive inhibitors for ATP. The kinase specificity of these inhibitors was investigated on more than 30 highly purified kinases: most kinases are not significantly inhibited; only the cell cycle regulating cdc2/cyclin B, cdk2/cyclin A and cdk2/cyclin E kinases, the brain cdk5/p25 kinase and ERK1 are substantially inhibited by olomoucine and roscovitine. Structure-activity relationship studies allow speculations on the interactions of olomoucine and roscovitine with the kinase catalytic subunit. Analysis of olomoucine/cdk2 and roscovitine/cdk2 co-crystal structures confirms that olomoucine and roscovitine bind in the ATP-binding pocket of cdk2, but shows that the purine rings of olomoucine/roscovitine and ATP are located in a totally different orientation. The anti-mitotic effects of olomoucine and roscovitine were investigated in a large variety of cellular models. The compounds inhibit both G1/S and G2/M transitions. The effects of cyclin-dependent inhibitors on apoptosis will be reviewed. We believe that the use of cell cycle control proteins provides highly specific mechanism-based screens for the discovery of anti-mitotic drugs of potential anti-tumour activity.

Meijer, L., 1996. Chemical inhibitors of cyclin-dependent kinases. **Trends in Cell Biology 6**, 393-397.

### The BACR/Zeneca Young Scientist of the Year Award Lecture

MOLECULAR CONTROL OF CELLULAR INVASION IN COLORECTAL CANCER CELLS: THE ROLE OF TYROSINE KINASES.

VG Brunton*[1], C Paraskeva[2] and MC Frame[1], [1]The Beatson Institute for Cancer Research, Switchback Road, Bearsden, Glasgow, G61 1BD and [2]Department of Pathology and Microbiology, University of Bristol, Bristol, BS8 1TD.

We have identified potential mediators of the behavioural changes which accompany the development of colon cancer. In particular, the epidermal growth factor (EGF) receptor and the focal adhesion kinase ($pp125^{FAK}$), molecules which are known to functionally co-operate with the Src family kinases, are elevated during the conversion of a pre-malignant colon adenoma cell line to a malignant colon carcinoma cell line *in vitro*. EGF induced the movement of the carcinoma cells into reconstituted basement membrane Matrigel in an *in vitro* invasion assay which was not seen with the premalignant adenoma cells. EGF did not alter the growth of either cell line. The increased motility was accompanied by an EGF-induced relocalisation of c-Src to discrete areas at the cell periphery and tyrosine phosphorylation of $pp125^{FAK}$ in the carcinoma cells but not the adenoma cells, suggesting that these tyrosine kinases are components of a pathway whose mis-regulation may contribute to colon cancer cell invasion. We also have a series of cell lines derived from a colon adenocarcinoma and lymph and hepatic metastases, taken from the same patient. In a wound healing assay hepatocyte growth factor (HGF) induced the migration of the metastatic cells into the wound but not those from the primary adenocarcinoma. This was dependent on the presence of extracellular matrix components and was not due to proliferation at the wound edge. The HGF-induced movement of the metastatic cells could be blocked by treatment with a Src tyrosine kinase inhibitor PD 162531, giving further support for a role for Src kinases in growth factor-induced cell motility. We are currently using both molecular and pharmacological agents to dissect further the pathways involved in growth factor-induced colon tumour cell motility and invasion.