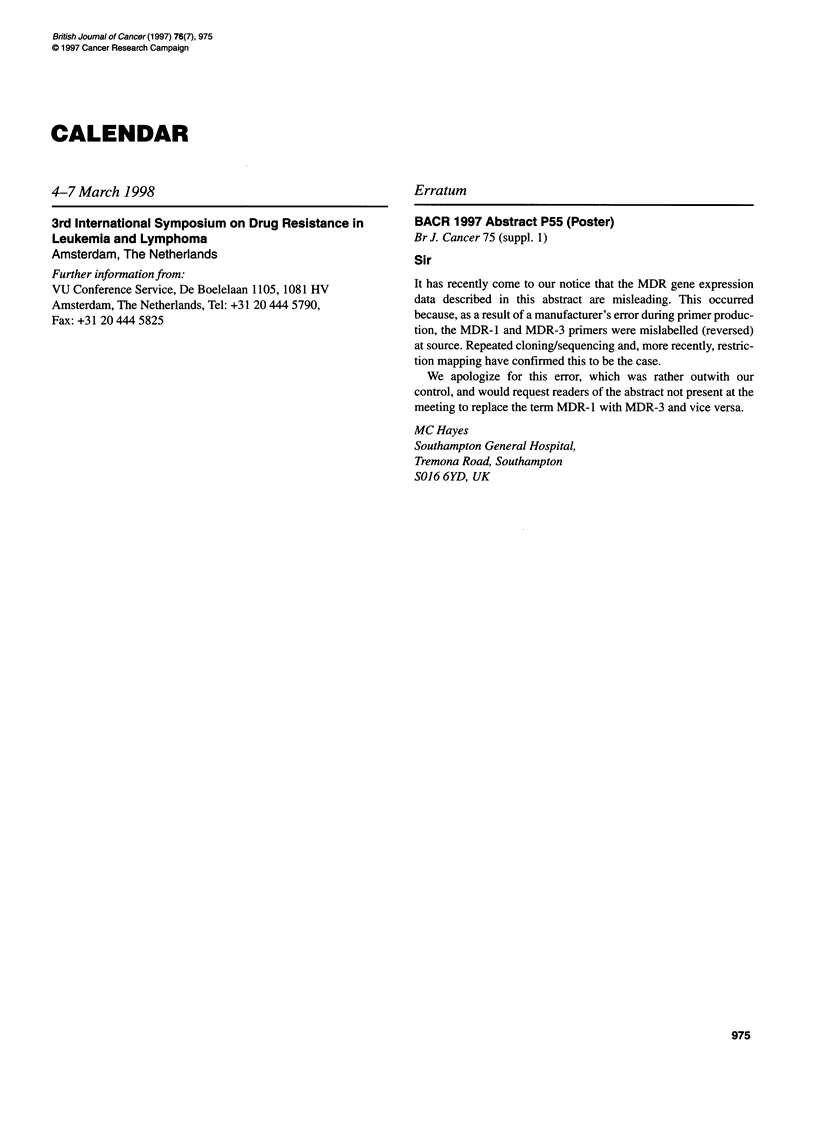# BACR 1997 Abstract P55 (Poster)

**Published:** 1997

**Authors:** 


*Erratum*

**BACR 1997 Abstract P55 (Poster)**
*Br J. Cancer* 75 (suppl. 1)

**Sir**

It has recently come to our notice that the MDR gene expression data described in this abstract are misleading. This occurred because, as a result of a manufacturer's error during primer production, the MDR-1 and MDR-3 primers were mislabelled (reversed) at source. Repeated cloning/sequencing and, more recently, restriction mapping have confirmed this to be the case.

We apologize for this error, which was rather outwith our control, and would request readers of the abstract not present at the meeting to replace the term MDR-1 with MDR-3 and vice versa.

*MC Hayes*
*Southampton General Hospital,*
*Tremona Road, Southampton*
*S016 6YD, UK*